# RBMS2 Chemosensitizes Breast Cancer Cells to Doxorubicin by Regulating BMF Expression

**DOI:** 10.7150/ijbs.66480

**Published:** 2022-02-07

**Authors:** Feng Xu, Tian Xia, Qi-Tong Xu, Xu Zhang, Yu-Zhou Huang, Xi Sun, Liang Shi, Xu-Jie Zhou, Ji-Fu Wei, Qiang Ding

**Affiliations:** 1 Jiangsu Breast Disease Center, the First Affiliated Hospital with Nanjing Medical University, 300 Guangzhou Road, Nanjing, 210029, China.; 2 Department of General Surgery, Comprehensive Breast Health Center, Ruijin Hospital, Shanghai Jiao Tong University School of Medicine Shanghai, 200032, China.; 3 Department of Breast Surgery, Fudan University Shanghai Cancer Center, No. 270, Dongan Road, Shanghai, 200032, China.; 4 Department of Pharmacy, Jiangsu Cancer Hospital & Jiangsu Institute of Cancer Research & The Affiliated Cancer Hospital of Nanjing Medical University, Nanjing, 210029, China.

**Keywords:** RBMS2, BMF, apoptosis, chemosensitization, doxorubicin, breast cancer

## Abstract

Chemoresistance is closely related to the therapeutic effect and prognosis in breast cancer patients. Increasing evidences demonstrated that RNA binding proteins (RBPs) have notable roles in regulating cancer cell proliferation, metastasis and chemotherapeutic sensitivity. RNA binding motif single stranded interacting protein 2 (RBMS2), an RBP, has been considered to be a tumor suppressor in several cancers. However, its role of doxorubicin sensitivity in breast cancer patients has not yet been fully revealed. Here, we performed doxorubicin cytotoxicity assay, flow cytometry and mouse xenograft model to examine the influence of RBMS2 on doxorubicin sensitization *in vitro* and *in vivo*. RIP assay and dual-luciferase reporter assay were performed to explore the relationship between RBMS2 and BMF. Our data demonstrated that upregulation of RBMS2 in breast cancer cells could enhance sensitivity to doxorubicin and promote apoptosis in the presence of doxorubicin, while inhibition of RBMS2 showed an opposite trend. Moreover, this chemosensitizing effect of RBMS2 could be reversed by the inhibition of Bcl-2 modifying factor (BMF). RBMS2 positively regulated BMF expression and increased BMF-induced expression of (cleaved) caspase 3, (cleaved) caspase 9 and poly (ADP-Ribose) polymerase (PARP). These results uncovered a novel mechanism for RBMS2 in the sensibilization of doxorubicin, suggesting that RBMS2 may act as a potential therapeutic target for drug-resistant breast cancer.

## Introduction

The incidence of breast cancer rises rapidly over the years, ranking as the first common cancer among females worldwide, and thus posing a considerable threat to female health 1, 2. The current treatment strategies for breast cancer include surgery, chemotherapy, radiotherapy, targeted treatment and endocrine therapy 3. Adjuvant systemic chemotherapy remains the preferred treatment for some patients of early stage and is suggested to be the major treatment for those with advanced stage 4. The commonly used chemotherapeutic drugs includes doxorubicin (DOX), paclitaxel and cyclophosphamide 5. However, the efficiency of chemotherapy is greatly limited by primary and acquired drug resistance 6, 7.

DOX is a cytotoxic anthracycline antibiotic used in anti-tumor therapy by inducing cancer cells apoptosis 8-10, which is regarded as the foundation of chemotherapy for breast cancer 11. Cellular resistance to DOX often develops in breast cancer patients with recurrence and advanced stage. Its molecular mechanisms have not been fully revealed 12. Various genetic and epigenetic factors can attribute to resistance of chemotherapy 13-15. Most chemotherapeutic drugs kill tumor cells by inducing apoptosis, so the absence of proapoptotic protein or the overexpression of anti-apoptotic proteins leads to resistance to chemotherapeutic drugs 16. BMF (Bcl-2 modifying factor) appertains to the Bcl-2 protein family and functions as a pro-apoptotic factor that is correlated with various cellular activities, including chemosensitivity. For instance, YAP/TEAD/SLUG axis suppressed apoptosis through transcriptional repression of BMF 17.

RBPs play a pivotal role at the post-transcriptional levels by several mechanisms, including mRNA stabilization, polyadenylation, transport, translation and RNA splicing 18, 19. Previous studies showed that numerous RBPs are dysregulated in different cancers, causing change of drug sensitivity 20, 21. Kim *et al*. found that non-POU domain containing octamer binding (NONO), an RBP, was highly expressed in triple-negative breast cancer and directly interacted with STAT3 protein by elevating its mRNA stability and transcriptional activity, which contributed to DOX resistance 22. RBMS2, also known as SCR3, a member of RBPs family, was extracted via phenotypic complementation of cdc2 and cdc13 mutants of yeast 23. Our previous study has illustrated that RBMS2 could act as a tumor suppressor and enhance the expression of P21 mRNA via directly binding to its AU-rich element (AREs) of 3′-untranslated region (3′-UTR) in breast cancer 24. According to our results of RNA sequencing from the above study, we found BMF mRNA was significantly upregulated after overexpression of RBMS2. In addition, our data showed that RBMS2 could induce breast cancer cells apoptosis. Therefore, we hypothesized that RBMS2 could sensitize breast cancer cells to DOX via inducing apoptosis regulated by BMF expression.

## Methods

### Cell culture

Human breast cancer DOX resistance cell line MCF-7/DOX was kindly provided by Dr. Shui Wang (Nanjing Medical University, Nanjing, China); MCF-7 cell line was purchased from the American Type Culture Collection (ATCC, USA) and SUM 1315 provided by Dr. Stephen Ethier (University of Michigan, AnnArbor, MI, USA). These three cell lines were cultured in DMEM (Wisent, China) containing 10% FBS (Gibco, USA), 4.5 mg/ml glucose, 100 μg/ml streptomycin and 100 μg/ml penicillin (Hyclone, USA), in a humidified incubator containing 5% CO_2_ at 37 °C.

### Lentivirus transfection and small interfering RNA

Lentiviral constructs to overexpress RBMS2 (RBMS2), the negative control (NC), knockdown RBMS2 (sh1, sh2) and the scramble control (SCR) were purchased from GenePharma (Shanghai, China) and generated following the manufacturer's instructions as previous described 24. For recovery assays, cells were first transduced with overexpressed and control vectors of RBMS2, and then transduced with BMF-siRNA (GenePharma, Shanghai, China), p21-siRNA (GenePharma, Shanghai, China) and the nonspecific siRNA control. All sequences are listed in Table S1. Real-Time quantitative PCR (RT-qPCR) and western blot were used to detect the transfection efficiency.

### Cytotoxicity assay

Cells were inoculated in 96-well plates (5×10^3^ cells/well) and grew in complete medium containing different concentrations of DOX (MedChemExpress, USA), cisplatin (MedChemExpress, USA) and 5-fluorouracil (5-FU) (MedChemExpress, USA) for 24 h, respectively. The cell survival rate was further tested by CCK-8 kit (Dojindo, Japan) following the manufacturer's guidelines. For colony formation assay, cells were firstly seeded in six-well plates with 1×10^3^ cells/well and incubated with DOX (0 or 1 μg/ml) for 24 h and then transferred to complete culture without DOX for 14 days. 1 μg/ml DOX was employed to keep the maximum inhibition rate below 50%. The resulting colonies were fixed in paraform for 20 min and stained with crystal violet solution for 20 min, then the colonies were counted and scanned.

### Apoptosis assay

Apoptosis was tested by the apoptosis kit (Annexin V-APC/7-AAD Apoptosis Detection Kit, Multisciences, China) based on the manufacturer's protocols. Briefly, cells were digested by EDTA-free trypsin and washed twice with ice cold phosphate buffered saline (PBS) and centrifuged (1200 rpm,5 min). Then, 400 μl annexin binding buffer (1×10^6^ cells/ml) was used to resuspend the cells. Finally, cells were dual stained using 5 μl APC-conjugated Annexin V and 10 μl 7-AAD and detected by flow cytometry (BD Biosciences, USA). Data were analyzed using FlowJo v10.0 (FlowJo, LLC).

### RT-qPCR

Total RNAs were isolated by Trizol reagent (TaKaRa, Japan) and HiScript qRT SuperMix (Vazyme, China) was used for complementary DNA (cDNA) synthesis. Thirty pairs of snap-frozen breast cancer tumor and adjacent tissues were collected in the First Affiliated Hospital of Nanjing Medical University from 2006 to 2016, and approved by the ethics and research committee of the First Affiliated Hospital with Nanjing Medical University. All included patients provided written informed consents. RT-qPCR was conducted using AceQ qPCR SYBR Green Master Mix (Vazyme, China) in a real-time PCR instrument (Roche, USA) following the manufacturer's instructions. The 2^-ΔΔCt^ method was calculated for relative mRNA expression and β-actin was utilized as the endogenous control. The sequences of primers used are listed in Table S2.

### Western blot analysis

The total protein extracted from breast cancer cells was split with RIPA buffer (P0013C, Beyotime, China) containing 1% phosphatase inhibitor, 1% PMSF, and 0.1% protease inhibitor and separated using 10% sodium dodecyl sulfate-polyacrylamide gel electrophoresis (SDS-PAGE), then electransferred onto an activated polyvinylidene fluoride (PVDF) membrane (Millipore, USA). Membranes were placed in Tris-buffered saline containing 0.1% Tween 20 (TBST) and 5% nonfat milk for 2 h and then incubated with primary antibodies against RBMS2 (1:1000, RayBiotech, USA, 102-12993), BMF (1:1000, Proteintech, China, 18298-1-AP), β-actin (1:1000, Proteintech, China, 20536-1-AP), Cleaved Caspase 3 (1:1000, Cell Signaling Technology, USA, 9664), Caspase 3 (1:1000, Cell Signaling Technology, USA, 14220), Cleaved Caspase 9 (1:1000, Cell Signaling Technology, USA, 9509), Caspase 9 (1:1000, Cell Signaling Technology, USA, 9504) , PARP (1:1000, Cell Signaling Technology, USA, 9532) overnight at 4 °C. Membranes were washed for 15 min for 3 times with TBST and then incubated for 2 h with secondary antibodies (1:5000, Cell Signaling Technology, USA, 7074P2). The expression levels of target protein were detected through Immobilob™ Western Chemiluminescent HRP Substrate (Millipore, USA).

### RNA stability assays

MCF-7 and SUM 1315 cells were transferred into 6-well plates and added with actinomycin D (Act D, 5 μg/ml) at 0, 1, 2, 4, 6 and 8 h when the number of cells reached 3×10^5^. Then total RNAs were extracted and the RNA expression levels of BMF were checked by RT-qPCR.

### RNA immunoprecipitation (RIP)

MCF-7 and SUM 1315 cells (2×10^7^) were washed with PBS and lysed by Magna RIP RNA-Binding Protein Immunoprecipitation Kit (Millipore, USA) following manufacturer's protocol. Then rabbit polyclonal anti-RBMS2 (5μg) and non-immunized rabbit IgG was incubated overnight at 4 °C. After RNA purification, protein A/G magnetic beads were used for the RNA protein immunocomplexes. Then RT-qPCR was conducted to examine the levels of BMF transcripts in the RBMS2 or IgG immunocomplexes.

### Dual-luciferase reporter assay

MCF-7 and SUM 1315 cells were seeded evenly into a 24-well plate and co-transfected with 200ng of pGL3 reporter vectors carrying 3′-UTR or the corresponding AREs mutant region of BMF and 5 ng of pRL-TK (Promega, USA). The AREs mutant reporter converts the AUUUA motif in the BMF 3′-UTR into AGGGA. These cells were harvested after transfection for 48h, and dual luciferase reporter assay kit (Promega, USA) was performed to detect the luciferase activity in strict accordance with the manufacturer's instructions.

### Xenograft tumors in nude mice

All animal investigations were performed according to the guidelines of Use Committee of the Nanjing Medical University and guidelines of Institutional Animal Care. Forty-eight female BALB/c nude mice (aged 4-6 weeks, 18-22 g) were purchased from the Experimental Animal Center of Nanjing Medical University (Nanjing, China). For subcutaneous inoculation, 1×10^6^ SUM 1315 cells stably expressing RBMS2 and the negative control (NC) resuspended in 100 μl PBS were implanted subcutaneously into the right flank regions of the mice. When the subcutaneous tumors reached to an average size of 0.5-0.6 cm (approximately 2 weeks after cell injection), the nude mice were randomly divided into four groups, namely, the NC-DOX, RBMS2-DOX, NC+DOX and RBMS2+DOX group. Then, all nude mice received either 2 mg/kg of DOX or equal volume of PBS by tail vein injection every 3 days and the growth of tumors was recorded every week by a caliper, calculated as (length × width^2^)/2. The mice were sacrificed at the end of experiments and the subcutaneous tumors were dissected out and photographed.

### Statistical analysis

GraphPad Prism (Version 8.0) was used to analyze and present the data. Significance of differences between groups was analyzed by Student′s t-test. Differenced expression profile of BMF in breast cancer and paired normal tissues was compared by the paired Student's t test. All experiments were independently repeated for three times, and p < 0.05 was used as statistically significant.

## Results

### RBMS2 could sensitize breast cancer cells to DOX *in vitro*

**Figure 1:**
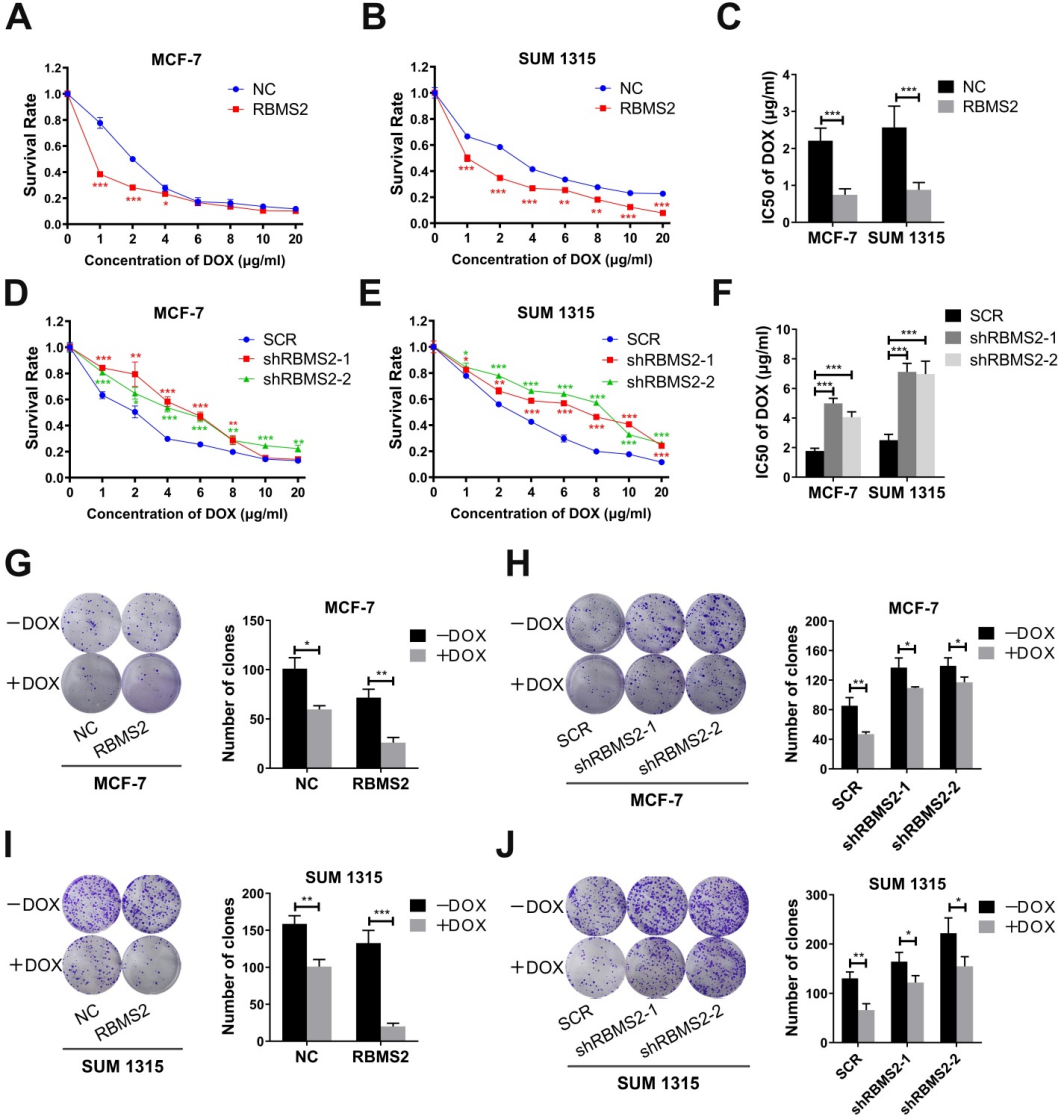
** RBMS2 could sensitize breast cancer cells to DOX *in vitro.*** RBMS2 overexpression and knockdown cell lines were treated with different dosages of DOX for 24 h, respectively. CCK-8 assay was used to examine the cell viability (**A and D** for MCF-7, **B and E** for SUM 1315) and IC50 value **(C and F)** of DOX. Transfected MCF-7 **(G, H)** and SUM 1315 **(I, J)** cells were treated with DOX (0 or 1 µg/ml) for 2 weeks. Representative images (left panel) and quantification (right panel) of colonies in the colony formation assay. Data were shown as mean ± SD. *p <0.05, **p <0.01, ***p<0.001.

Overexpression and knockdown of RBMS2 in MCF-7 and SUM 1315 stable cells constructed by lentivirus were validated via RT-qPCR and western blot (Fig. 4G-J). RBMS2 acted as a tumor suppressor and has relatively low expression in breast cancer, resulting in the minor knockdown effects. The half-maximal inhibitory concentration (IC50 value) of DOX in RBMS2 overexpressing cells was decreased by 66.3% in MCF-7 cells (Fig. 1A) and 65.7% in SUM 1315 cells (Fig. 1B). On the contrary, knockdown of RBMS2 showed an opposite trend, with the IC50 values increasing to 182% and 129% in MCF-7 cells (Fig. 1D) and 185% and 179% in SUM 1315 cells (Fig. 1E) respectively. IC50 values were shown in Fig. 1C and Fig. 1F. Although the proliferation of RBMS2 knockdown and overexpression cell lines were both inhibited by DOX, the IC50 values of overexpression group dropped more significantly than that of knockdown group. These data suggested that knockdown of RBMS2 could affect the sensitivity of DOX in breast cancer cell lines. The same effect was also observed in MCF-7/DOX cell lines after overexpression of RBMS2, with IC50 values decreasing by 58.8% (Fig. S1). As demonstrated in Fig. 1G and Fig. 1I, after DOX treatment, the ability of RBMS2 overexpressed breast cancer cells to form colonies was much worse than that of control cells, while inhibition of RBMS2 impaired the proliferation of breast cancer to a much lesser extent (Fig. 1H, J). Collectively, RBMS2 sensitized breast cancer cells to DOX *in vitro*.

### RBMS2 could sensitize breast cancer cells to DOX *in vivo*

**Figure 2:**
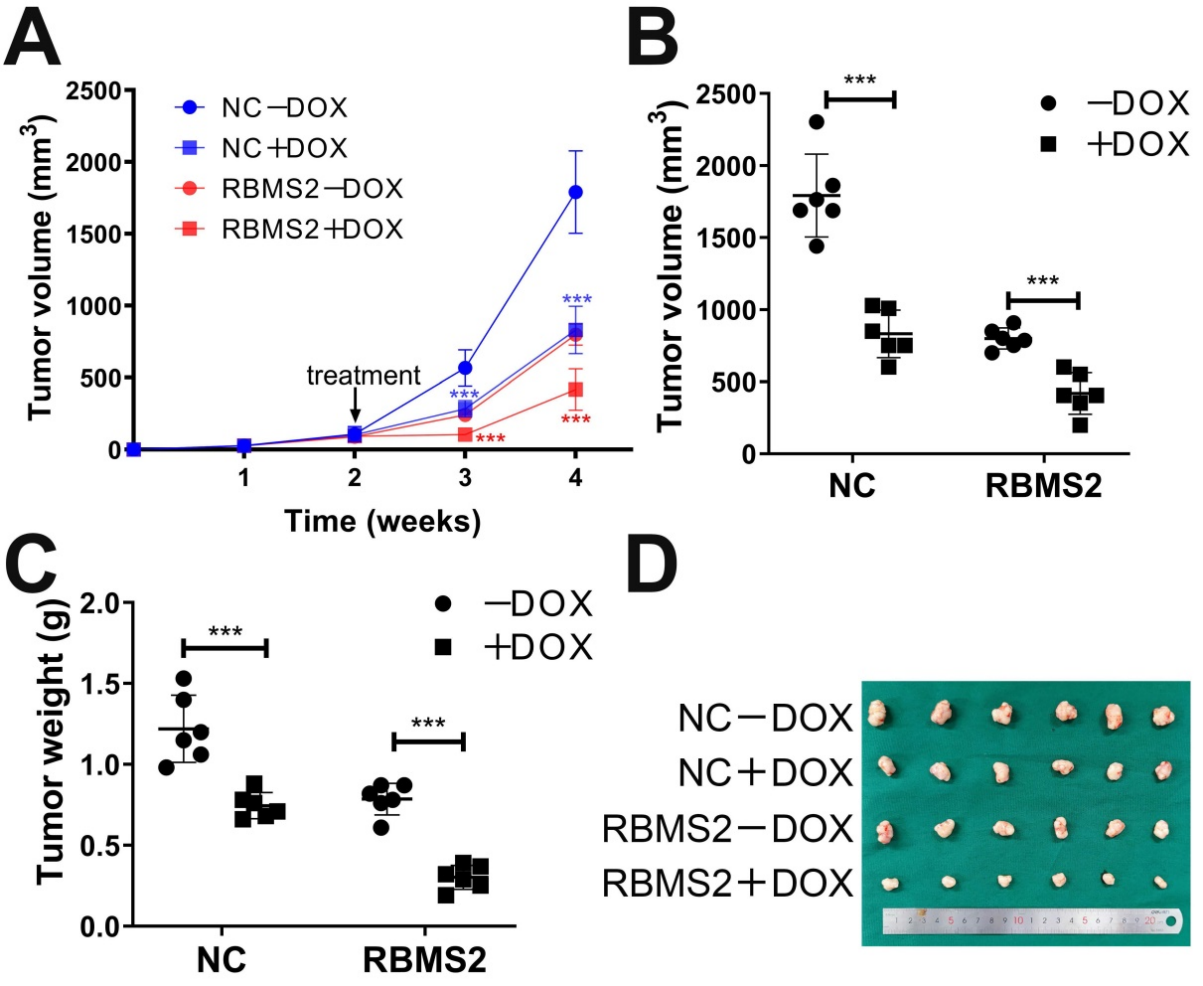
** Overexpression of RBMS2 could enhance the therapeutic effect of DOX *in vivo*.** Tumor volumes were measured in different treatment groups **(A. B)**. The excised tumor lumps were weighed **(C)** and photographed **(D)**. Data were shown as mean ± SD. *p <0.05, **p <0.01, ***p<0.001.

To extend our results on the influence of RBMS2 on DOX sensitivity in breast cancer, a mouse xenograft model was carried out by subcutaneous injection with RBMS2 overexpression stable cells or control cells. As expected, with or without DOX treatment, tumors derived from RBMS2 group proliferate and expand significantly slower than that of control group (Fig. 2A). As demonstrated in Fig. 2B, tumors volume of RBMS2 and DOX combination group was relatively smaller than that of NC+DOX group. 4 weeks later, tumor weights in RBMS2+DOX were remarkably lighter than that of other groups, which indicated RBMS2 sensitized breast cancer cells to DOX *in vivo* (Fig. 2C-D).

### RBMS2 could induce breast cancer cells apoptosis

**Figure 3:**
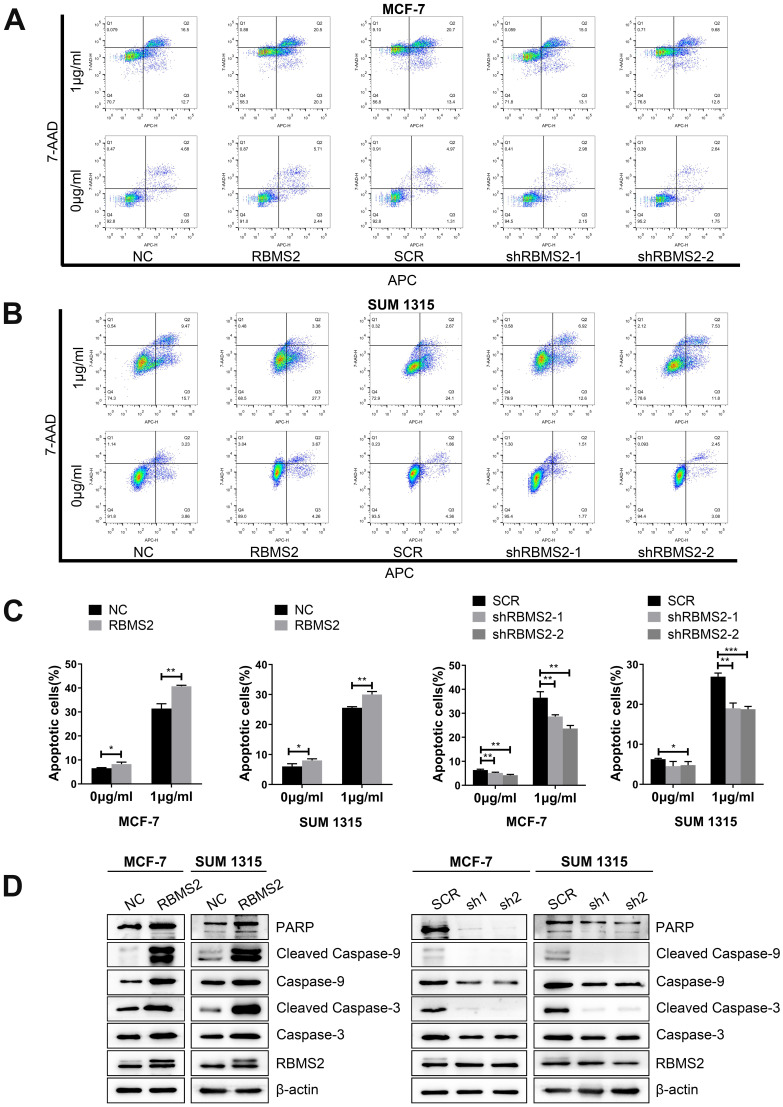
** RBMS2 could induce apoptosis and apoptosis related proteins in breast cancer cell lines.** Flow cytometry analysis of apoptosis in RBMS2 overexpression and knockdown MCF-7 and SUM 1315 cells with or without DOX treatment **(A, B)**. The statistical calculation of apoptotic rates was shown **(C)**. The levels of (cleaved) caspase 3, (cleaved) caspase 9 and PARP in MCF-7 and SUM 1315 cells transfected with RBMS2 overexpression and knockdown were detected by Western blot **(D)**. The top band of RBMS2 is the exogenously band in western blot. Data were shown as mean ± SD. *p <0.05, **p <0.01, ***p<0.001.

The effects of RBMS2 on apoptosis in MCF-7 and SUM 1315 cells were detected by flow cytometry. Overexpression of RBMS2 robustly strengthened DOX-induced apoptosis in breast cancer cells, while inhibition of RBMS2 abated DOX-induced apoptosis (Fig. 3A-B). Apoptosis ratios were shown in Fig. 3C. Then, western blotting was performed to investigate the expression of apoptosis-related proteins after the expression of RBMS2 was altered. The target protein expressions were consistent between NC and SCR groups in western blot analysis. The levels of the pro-apoptosis protein (cleaved) caspase 3, (cleaved) caspase 9 and PARP were increased compared to that of control group after the overexpression of RBMS2. In particular, cleaved caspase 3 and cleaved caspase 9 were more significantly upregulated than caspase 3 and caspase 9, indicating that RBMS2 could induce breast cancer cells apoptosis. In contrast, knockdown of RBMS2 showed an opposite trend (Fig. 3D). These data suggested RBMS2 could induce cell apoptosis and modulate apoptosis-related proteins in breast cancer cells.

### RBMS2 could elevate the expression of BMF in breast cancer cells

**Figure 4:**
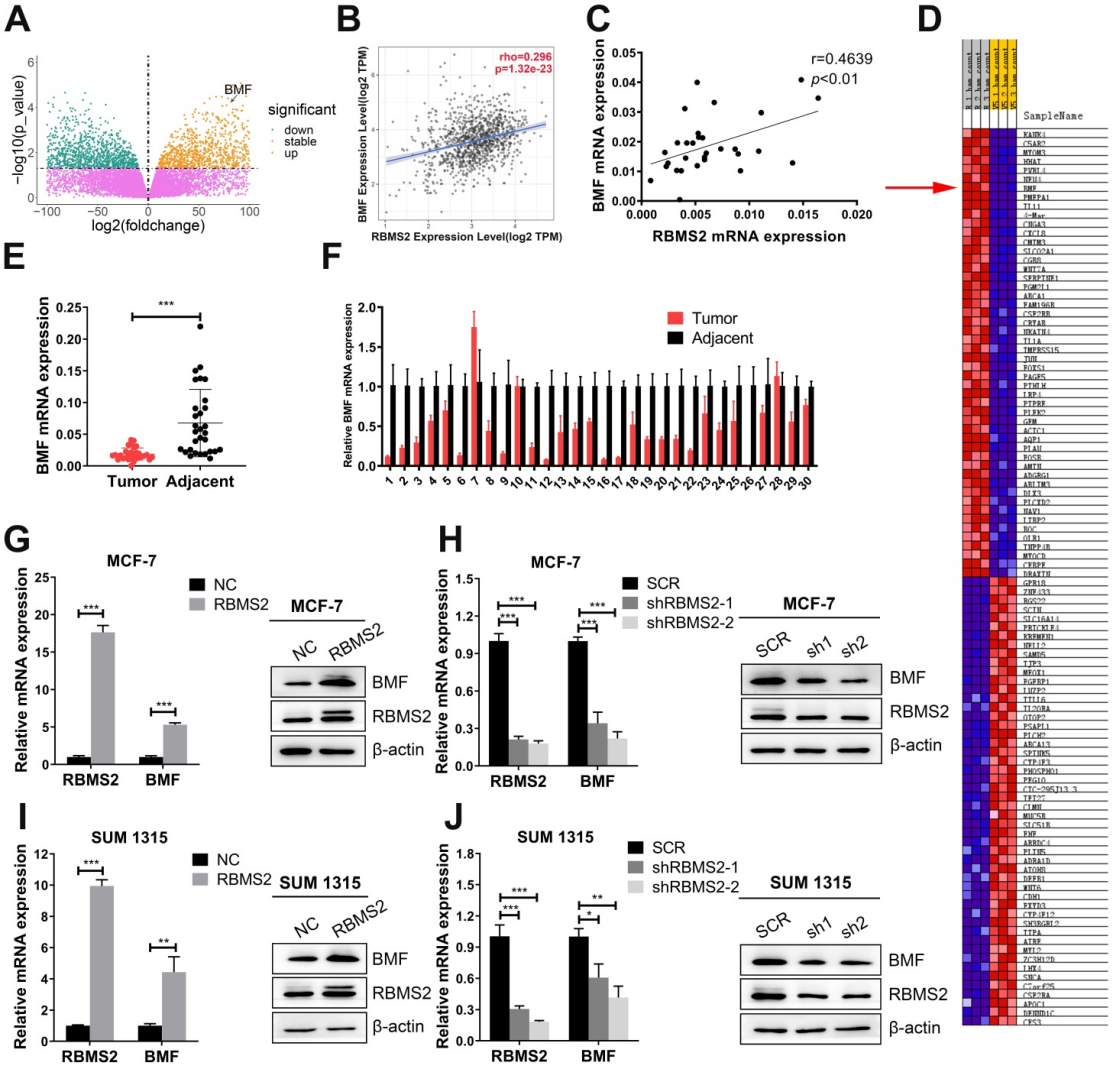
** RBMS2 regulated the expression of pro-apoptotic protein BMF.** Volcano plot represented the distribution of mapped transcripts **(A)**. The size and color of the dots meant the number of enriched genes and the adjusted p values, respectively. BMF was found to be positively correlated with RBMS2 in breast cancer both from TIMER (Tumor Immune Estimation Resource, https://cistrome.shinyapps.io/timer) database (B), the patients' samples from our hospital **(C)**. Correlation heatmap were used to analyze the correlation between RBMS2 and BMF **(D)**. R1, R2, R3 and V5-1, V5-2 and V5-3 represented RBMS2-overexpressed group and the control group, respectively. The red arrow indicated that BMF was positively correlated with RBMS2. Expression of BMF in breast cancer tissues and normal tissues **(E, F)**. Overexpression of RBMS2 significantly increased the expression of BMF at both mRNA and protein **(G, I)** levels. BMF was significantly down regulated after RBMS2 knock down at both mRNA and protein levels **(H, J)**. The top band of RBMS2 is the exogenously band in western blot. Data were shown as mean ± SD. *p <0.05, **p <0.01, ***p<0.001.

Previous RNA sequencing data of overexpressing RBMS2 and control group in SUM 1315 cells revealed that BMF might be a potential mRNA target mediated by RBMS2 24. BMF was dramatically upregulated as the expression of RBMS2 increased with log2 (foldchange) 2.35 and FDR < 0.05 (Fig. 4A). BMF was found to be positively correlated with RBMS2 in both TIMER (Tumor Immune Estimation Resource, https://cistrome.shinyapps.io/timer) database (Fig. 4B) and the breast cancer samples from our hospital (Fig. 4C). The same results were obtained for the correlation heatmap (Fig. 4D). Moreover, a remarkable decrease of BMF expression was reported in breast cancer tissues compared to that of paracancerous tissues (Fig. 4E-F). The expression of BMF was promoted after the MCF-7 (Fig. 4G) and SUM 1315 cells (Fig. 4I) were transfected with RBMS2 overexpressing lentivirus. Accordingly, the expression level of BMF in RBMS2 knockdown MCF-7 cells (Fig. 4H) and SUM 1315 cells (Fig. 4J) was significantly suppressed.

### RBMS2 could increase BMF mRNA stability

**Figure 5:**
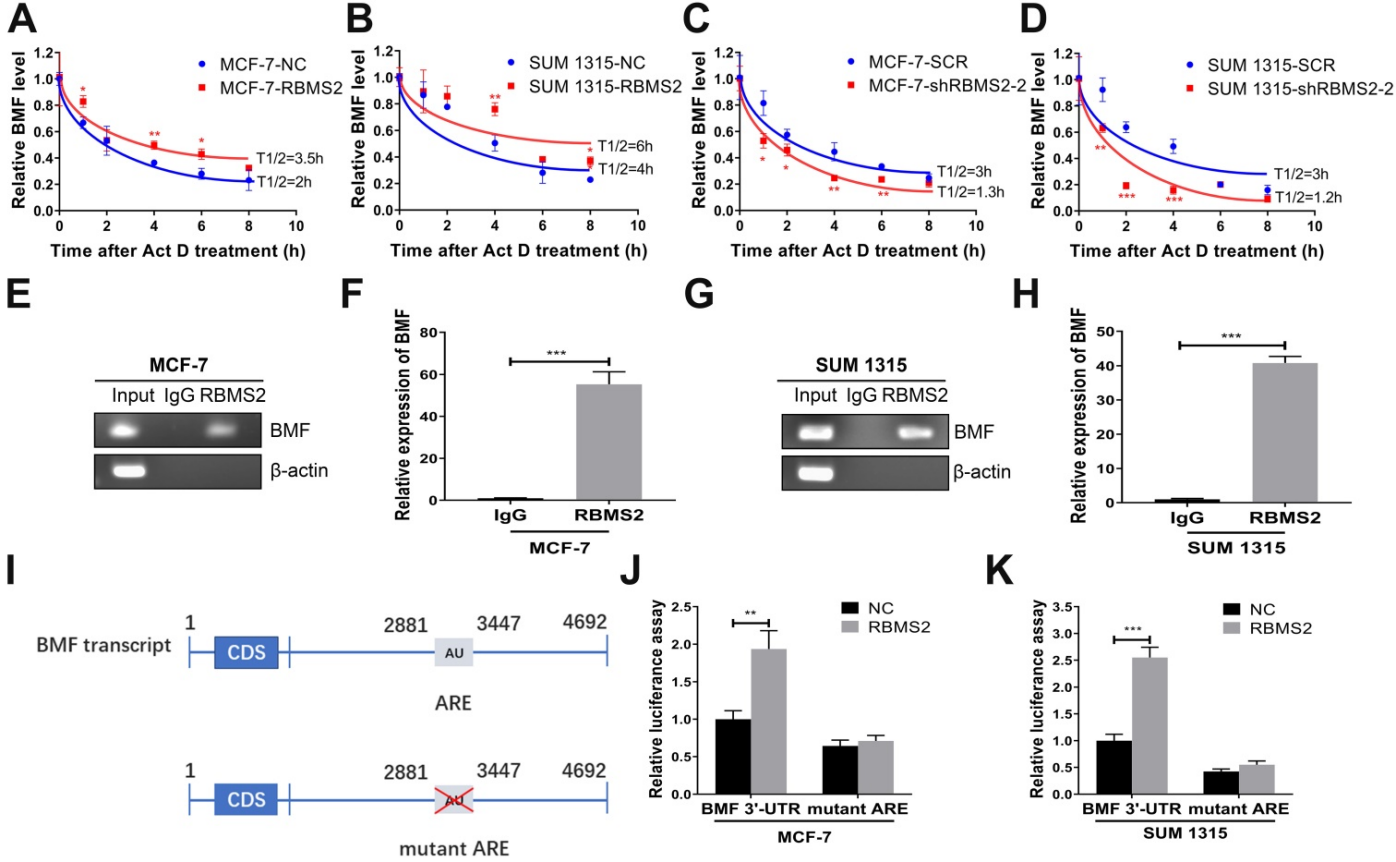
** RBMS2 could increase BMF mRNA stability.** In MCF-7 and SUM 1315 cell lines, RBMS2 overexpression prolonged the half-life of BMF mRNA after treated with Act D at a concentration of 5 µg/ml **(A, B)**, while knockdown of RBMS2 shortened the half-life of BMF mRNA **(C, D)**. MCF-7 and SUM 1315 cells lysates were immunoprecipitated with RBMS2 or IgG antibody and analyzed using PCR **(E, G)** and RT-qPCR **(F, H)** to detect transcript levels of BMF. Schematic diagram containing BMF 3'-UTR (upper) and AREs mutant region (lower) **(I)**. The reporter containing BMF 3'-UTR was increased after overexpression of RBMS2 in MCF-7 **(J)** and SUM 1315 **(K)** cell lines. Firefly luciferase activity was detected and normalized to Renilla luciferase activity. Data were shown as mean ± SD. *p <0.05, **p <0.01, ***p<0.001.

After 5 μg/ml Act D treatment at different time points (0-8 h), the half-life of BMF mRNA in RBMS2 overexpressing cells increased from 2 to 3.5h in MCF-7 cells (Fig. 5A) and 4 to 6 h in SUM 1315 cells (Fig. 5B). In contrast, the opposite result was found when RBMS2 was knocked down, with the half-life decreasing from 3 to 1.3 h in MCF-7 cells (Fig. 5C) and 3 to 1.2 h in SUM 1315 cells (Fig. 5D). All these results indicated that RBMS2 could enhance BMF expression by elevating its mRNA stability. To further dissect whether RBMS2 binds directly to the BMF mRNA in breast cancer cells, RIP assay was used in MCF-7 (Fig. 5E-F) and SUM 1315 (Fig. 5G-H) cells followed by RT-PCR and RT-qPCR. The BMF mRNA was presented in RBMS2 and Input, whereas not in IgG. β-actin, which used as a negative control that could not bind to RBMS2 mRNA. The results showed that RBMS2 could physically bind to BMF mRNA. Then, luciferase reporter assay which contained the whole region of BMF 3′-UTR or AREs mutant BMF 3′-UTR was used to further explore whether RBMS2 specifically regulated BMF via binding to the AREs in 3′-UTR of BMF mRNA (Fig. 5I). Our results suggested that RBMS2 could enhance the luciferase activity of a reporter containing the 3′-UTR of the BMF mRNA in comparison with control. On the contrary, the luciferase activity of a reporter containing ARE sites mutant BMF 3′-UTR in RBMS2 and control group was similar (Fig. 5J-K). These findings indicated that RBMS2 could bind directly to the ARE sites in the 3′-UTR of BMF mRNA, enhancing its expression and stability.

### Inhibition of BMF could reverse the sensitization to DOX induced by RBMS2 both *in vivo* and *in vitro*

**Figure 6:**
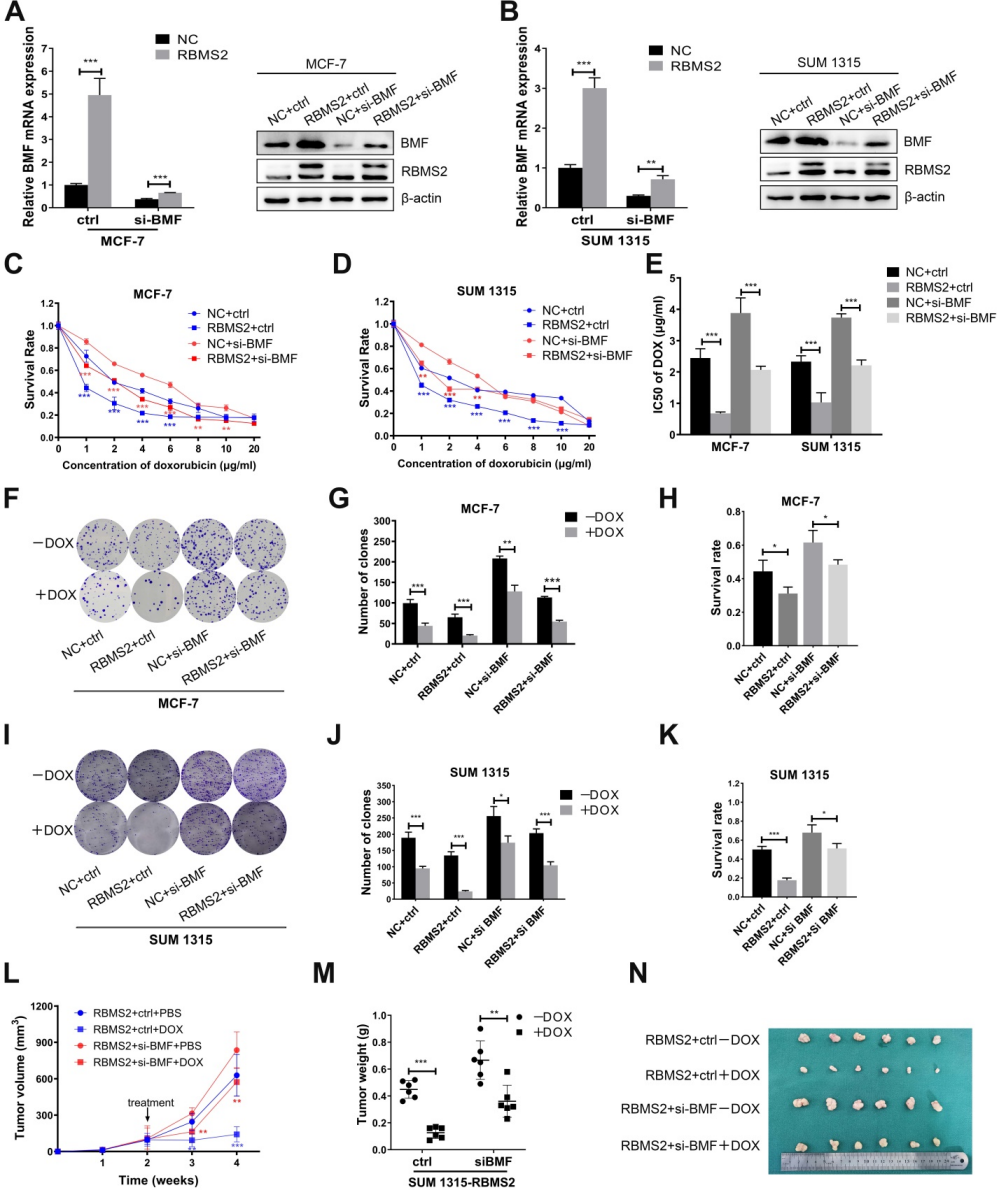
** BMF could reverse the sensitization to DOX induced by RBMS2 both *in vivo* and *in vitro*.** Small interfering RNA of BMF was transfected into RBMS2 overexpression MCF- 7 and SUM 1315 cells. Transfection efficiency was confirmed via RT-qPCR and western blot **(A, B)**. The top band of RBMS2 is the exogenously band in western blot. The sensitivity to DOX of MCF-7 and SUM 1315 cells mentioned above was examined using CCK-8 and colony formation. CCK-8 assay was used to examine cell viability (**C** for MCF-7, **D** for SUM 1315) and IC50 value **(E)** of DOX. Representative photographs **(F, I)**, quantification **(G, J)** and cell survival after DOX treatment **(H, K)** were shown by colony formation assay. Tumor volumes were measured in different treatment groups **(L)**. The excised tumor lumps were weighed **(M)** and photographed **(N)**. Data were shown as mean ± SD. *p <0.05, **p <0.01, ***p<0.001.

To explore whether the RBMS2 induced sensitization to DOX was depended on BMF expression, we performed rescue experiments. RBMS2-over-expressed MCF-7 and SUM 1315 cells were transfected with si-BMF or control. We set up four groups: NC+ctrl, NC+si-BMF, RBMS2+ctrl and RBMS2+si-BMF. RT-qPCR and western blot were performed to verify the transfection efficiency (Fig. 6A-B). IC50 value of DOX was reduced in the RBMS2-overexpressed groups compared with the NC group. However, on concurrent inhibition of BMF, this resistance to DOX reactivated significantly (Fig. 6C-E). Same trend was observed in colony formation assay (Fig. 6F-K) and tumor xenograft model (Fig. 6L-N).

### Inhibition of BMF could rescue the breast cancer cells apoptosis induced by RBMS2

**Figure 7:**
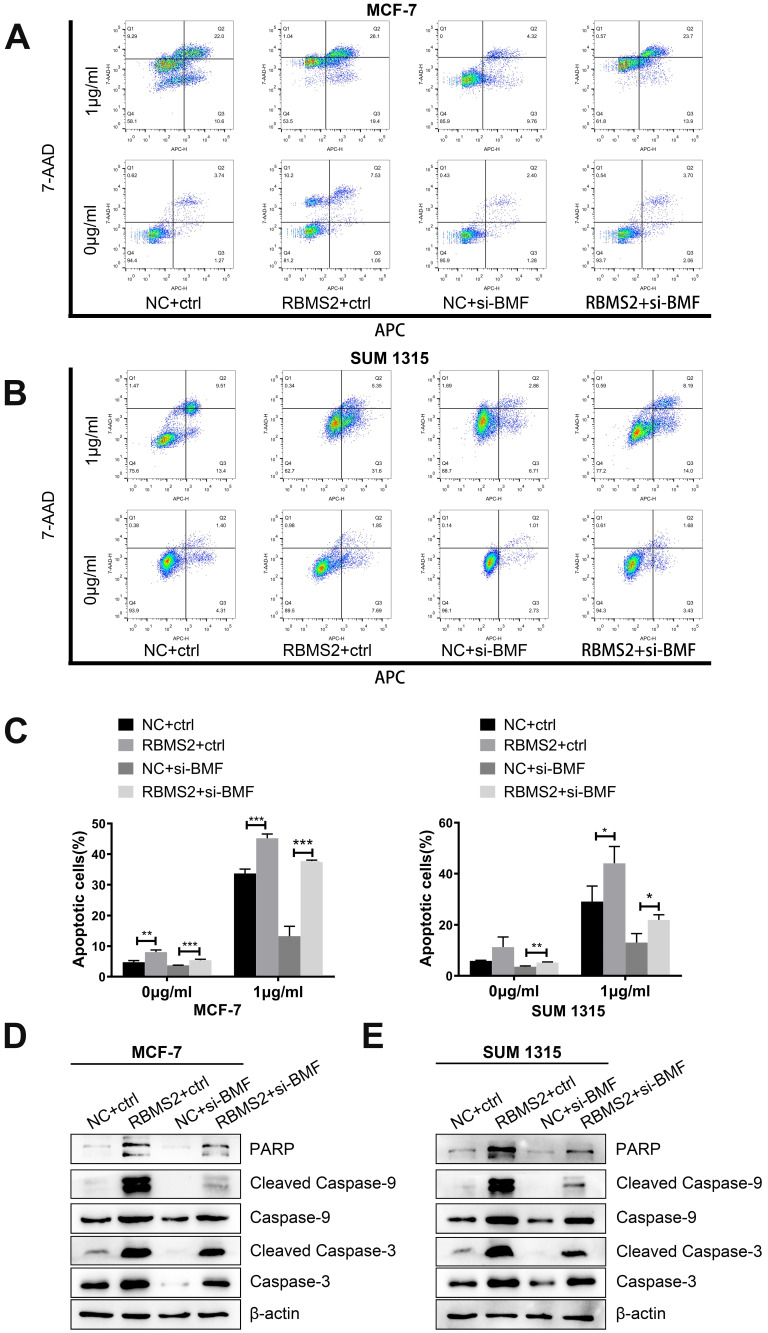
** BMF could rescue the apoptosis and apoptosis related proteins induced by RBMS2 in breast cancer cell lines.** Apoptosis and apoptosis related proteins were detected by flow cytometry analysis **(A, B, C)** and western blot **(D, E)**. Data were shown as mean ± SD. *p <0.05, **p <0.01, ***p<0.001.

To confirm whether the knockdown of BMF changes the increase in RBMS2-induced apoptosis rate in the presence of DOX, the apoptosis rate of all groups was measured by flow cytometry. RBMS2 overexpression dramatically increased the apoptosis under DOX treatment. But RBMS2+ si-BMF group dramatically decreased the apoptosis rate compared to the RBMS2+ctrl group (Fig. 7A-B). The apoptotic rates were shown in Fig. 7C. Western blotting showed the expression levels of (cleaved) caspase 3, (cleaved) caspase 9, (cleaved) caspase 3, (cleaved) caspase 9 and PARP were decreased after the attenuation of BMF. The increased ratio in cleaved caspase 3/caspase 3 and cleaved caspase 9/caspase 9 were partly reversed by the attenuation of BMF (Fig. 7D-E). Our previous study identified p21 as another key target gene mediating the tumor suppressing role of RBMS2 24. However, depletion of p21 could not reverse the sensitization to DOX induced by RBMS2 (Fig. S2). In summary, BMF could rescue the apoptosis of breast cancer cells induced by RBMS2.

## Discussion

Our results revealed the unique mechanism on how RBMS2 could contribute to antineoplastic signaling. RBMS2 could promote BMF mRNA stability by binding AREs in the BMF mRNA 3′-UTR. In our study, we elucidated that RBMS2, together with BMF, could enhance apoptosis of breast cancer cells to promote the sensitivity to DOX.

Resistance to apoptosis is considered an important hallmark of cancer, and all chemical treatments are believed to achieve their function directly or indirectly through inducing cell apoptosis 25. And inducing apoptosis has been used as a logical and realistic therapeutic strategy since the 1980s 26. Our previous study illustrated that gene regulations mediated by RBPs are closely correlated with numerous cancer-related biological functions, such as proliferation and epithelial-mesenchymal transition (EMT) 27-29. As overexpression of RBMS2 could enhance susceptibility to DOX in MCF-7/DOX cell lines, we hypothesized that, its ectopic expression might result in drug resistant phenotype by enhancing the cellular sensitivity to DOX. In this study, we found that overexpression of RBMS2 could improve DOX sensitivity and induce apoptosis in breast cancer cells, while inhibition of RBMS2 showed an opposite effect. We hypothesized that RBMS2 could enhance the sensitivity of DOX by inducing apoptosis, considering the resistance mechanism of DOX. Hence, apoptosis-related molecules were in the spotlight. According to our previous results of mRNA sequencing 24, we found that mRNA level of BMF was significantly upregulated in breast cancer cells. In our study, BMF was remarkably enhanced after RBMS2 overexpression. In general, BMF is associated with diverse cellular activities, including apoptosis and chemosensitivity 30, 31. Various studies showed that overexpression of BMF could activate the release of BIM from BCL-XL to induce mitochondrial apoptosis mediated by (cleaved) caspase 3, (cleaved) caspase 9 and PARP 32, 33. Then, we discovered that BMF expression was downregulated and positively correlated with RBMS2 expression in breast cancer and normal tissues from our hospital and TCGA databases. In our study, the expression of BMF and its effectors including (cleaved) caspase 3, (cleaved) caspase 9 and PARP were all enhanced by overexpression of RBMS2, resulting in the induction of apoptosis. And the sensibilization of DOX mediated by RBMS2 could be interfered by attenuating the expression of BMF, suggesting BMF was a primary target of RBMS2. Therefore, we concluded that RBMS2 regulated BMF, which could enhance the expression of (cleaved) caspase 3, (cleaved) caspase 9 and PARP, affecting the cellular sensitivity to DOX by promoting apoptosis. Knockdown of BMF alone in the absence of DOX could increase cell growth both *in vitro* and *in vivo*. The depletion of BMF may be a cause of resistance to DOX. Nevertheless, RBMS2-BMF axis could not sensitize breast cancer cells to 5-FU or cisplatin (Fig. S3). Further animal experiments confirmed the sensibilization to DOX of RBMS2 in breast cancer cells. Under the same dosage of DOX, tumors formed in nude mice with RBMS2-over-expressed SUM 1315 were more sensitive than the control group, consisting with the results *in vitro*. In our study, the same conclusions were confirmed both in ER^+^ and triple-negative breast cancer lines. However, whether there is a pervasive influence in different sub-types of breast cancer is unclear. Considering the few studies on RBMS2, further research will be conducted in other types of breast cancer.

In this study, overexpression of RBMS2 could increase BMF mRNA stability, as detected by RNA stability assays. RIP assay and dual-luciferase reporter assay also demonstrated that RBMS2 could bind directly to the AREs in 3′-UTR of BMF mRNA, leading to increased expression of (cleaved) caspase 3, (cleaved) caspase 9 and PARP, which were considered to be effectors of BMF and therefore contributed to higher apoptosis rate 34. All the above data suggested RBMS2 could regulate the expression of BMF via a posttranscriptional regulation, which is the most common way for RBPs 35, 36. Summarily, our study reveals a novel mechanism for RBMS2 as a regulator of chemotherapy response to DOX in breast cancer.

## Conclusions

Here, our study first reported that RBMS2 could induce apoptosis and increase DOX sensitivity by elevating BMF expression in breast cancer cells. Mechanistically, RBMS2 could stabilize the mRNA of BMF by directly interacting with the AREs in the 3′-UTR. Activating RBMS2 expression may represent a novel strategy for drug-resistant breast cancer.

## Supplementary Material

Supplementary figures and tables.

## Abbreviations

RBPs: RNA binding proteins
RBMS2: RNA binding motif single stranded interacting protein 2
BMF: Bcl-2 Modifying Factor
PARP: poly (ADP-Ribose) polymerase
RT-qPCR: Real-time quantitative polymerase chain reaction
cDNA: Complementary DNA
3′-UTR: 3-untranslated region
AREs: AU-rich element
RIP: RNA immunoprecipitation
