# Supplementary material for: RBMS2 Chemosensitizes Breast Cancer Cells to Doxorubicin by Regulating BMF Expression
Source: Int J Biol Sci. 2022 Feb 7;18(4):1724–36. doi: 10.7150/ijbs.66480 (PMC8898364; doi:10.7150/ijbs.66480)
Supplement: Supplementary file 1 — Supplementary figures and tables. [file ijbsv18p1724s1.pdf]

Table S1: The sequences of the siRNAs

Si-BMF	Sense	5′-GCACAACCUUGCUUUGAAUTT-3′
Si-BMF	Antisense	5′-AUUCAAAGCAAGGUUGUGCTT-3′
Si-p21	Sense	5′-CCUCUGGCAUUAGAAUUAUTT-3′
Si-p21	Antisense	5′-AUAAUUCUAAUGCCAGAGGTT-3′

Table S2: The primers used in RT-qPCR

β-actin	Forward	5′-GCTGTGCTATCCCTGTACGC-3′
β-actin	Reverse	5′-TGCCTCAGGGCAGCGGAACC-3′
RBMS2	Forward	5′-AGTTCTGACACCAGGGATGG-3′
RBMS2	Reverse	5′-TGCTCCTCGACTGAAACA-3′
BMF	Forward	5′-AGGTACAGATTGCCCGAAAG-3′
BMF	Reverse	5′- TTCCTGTTCCAGACGGTGTT-3′
p21	Forward	5′-TGTCCGTCAGAACCCATGC-3′
p21	Reverse	5′-AAAGTCGAAGTTCCATCGCTC-3′

Figure S1

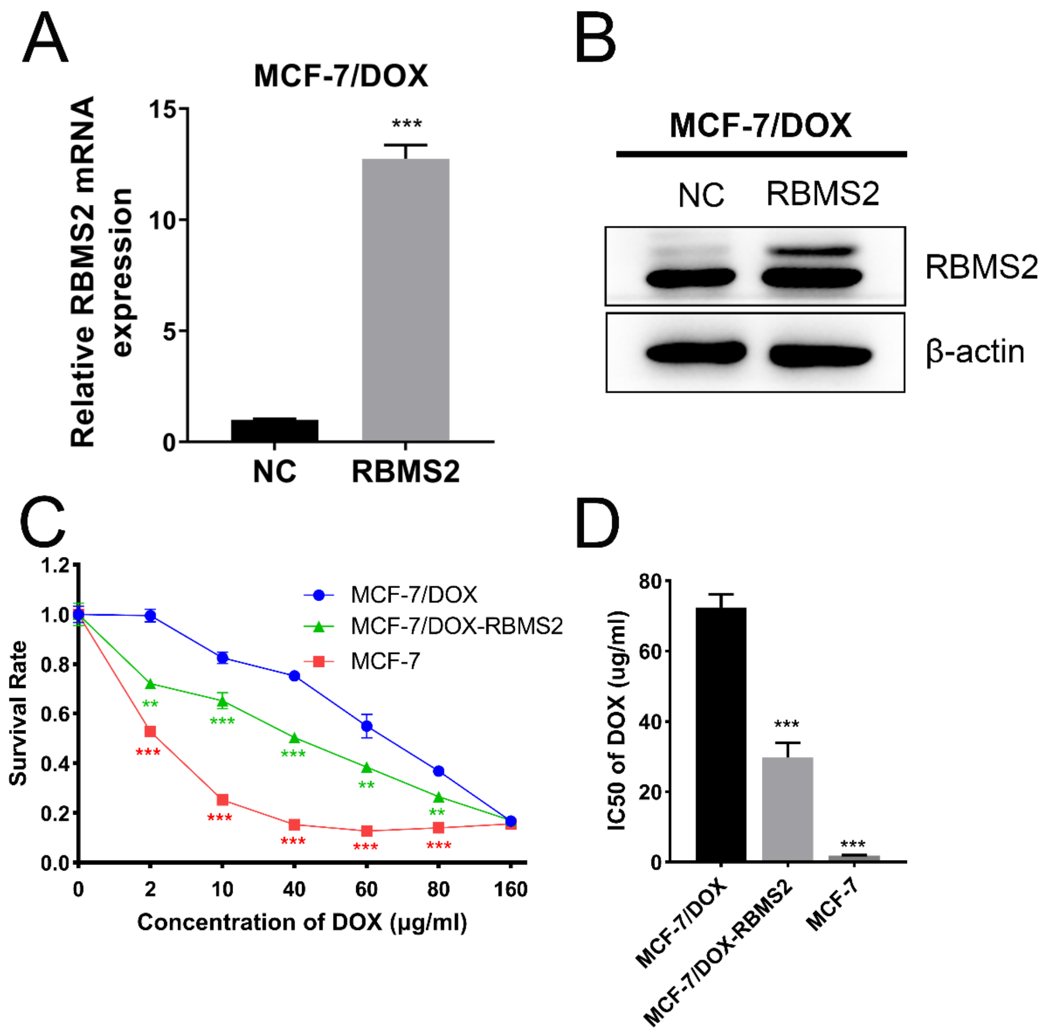
A
MCF-7/DOX
Relative RBMS2 mRNA expression
15
10
5
0
***
NC
RBMS2
B
MCF-7/DOX
NC
RBMS2
RBMS2
β-actin
C
Survival Rate
1.2
1.0
0.8
0.6
0.4
0.2
0.0
MCF-7/DOX
MCF-7/DOX-RBMS2
MCF-7
**
***
***
***
***
**
**
***
***
***
***
0
2
10
40
60
80
160
Concentration of DOX (μg/ml)
D
IC50 of DOX (ug/ml)
80
60
40
20
0
***
***
MCF-7/DOX
MCF-7/DOX-RBMS2
MCF-7

Figure S2

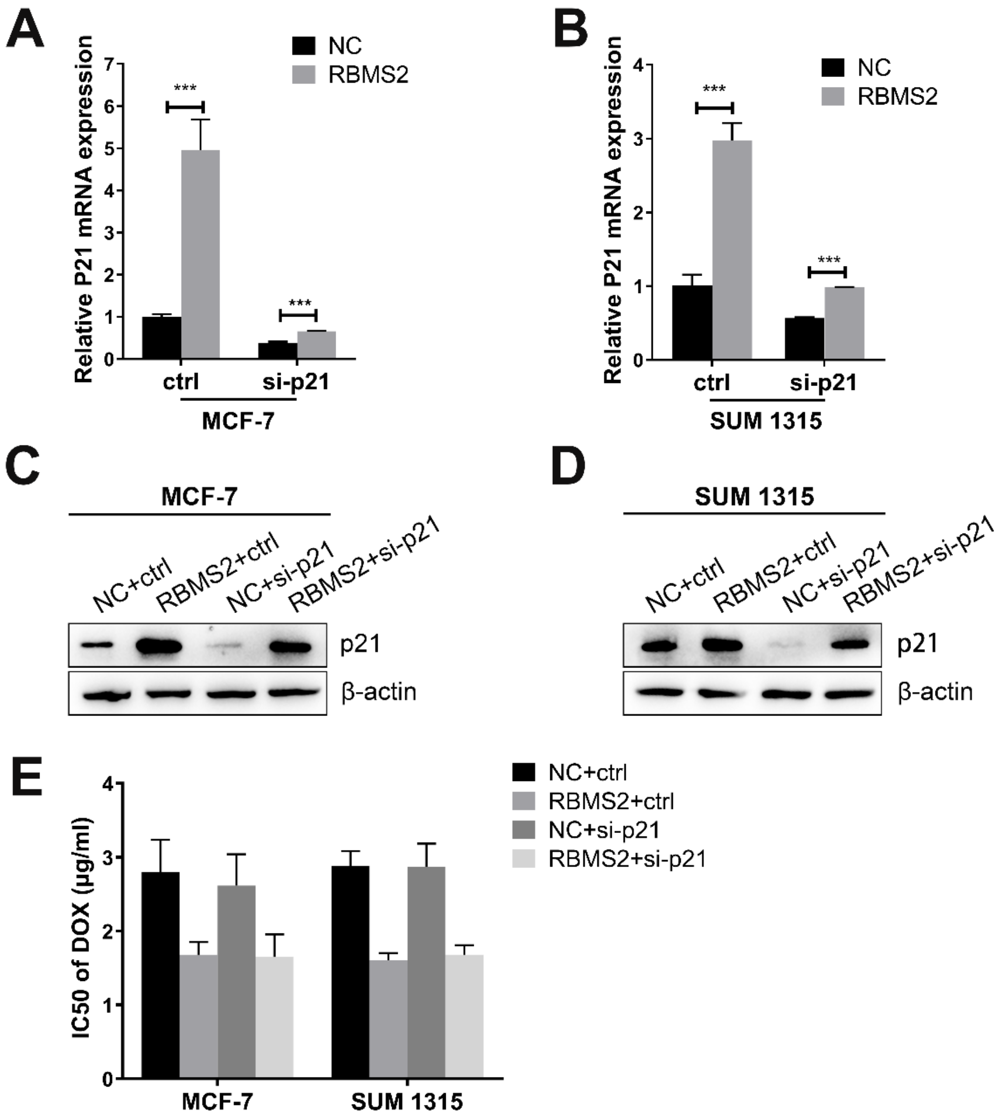
A
NC
RBMS2
Relative P21 mRNA expression
7
6
5
4
3
2
1
0
***
***
ctrl
si-p21
MCF-7
B
NC
RBMS2
Relative P21 mRNA expression
4
3
2
1
0
***
***
ctrl
si-p21
SUM 1315
C
MCF-7
NC+ctrl
RBMS2+ctrl
NC+si-p21
RBMS2+si-p21
p21
β-actin
D
SUM 1315
NC+ctrl
RBMS2+ctrl
NC+si-p21
RBMS2+si-p21
p21
β-actin
E
NC+ctrl
RBMS2+ctrl
NC+si-p21
RBMS2+si-p21
IC50 of DOX (μg/ml)
4
3
2
1
0
MCF-7
SUM 1315

Figure S3

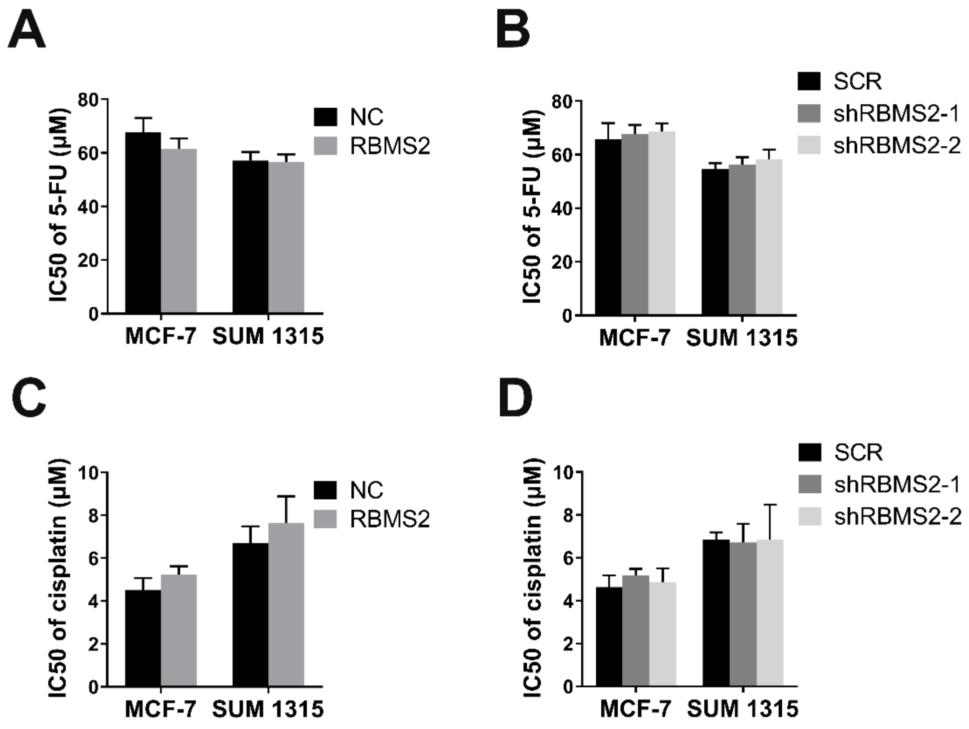
A
IC50 of 5-FU (μM)
80
60
40
20
0
MCF-7
SUM 1315
NC
RBMS2
B
IC50 of 5-FU (μM)
80
60
40
20
0
MCF-7
SUM 1315
SCR
shRBMS2-1
shRBMS2-2
C
IC50 of cisplatin (μM)
10
8
6
4
2
0
MCF-7
SUM 1315
NC
RBMS2
D
IC50 of cisplatin (μM)
10
8
6
4
2
0
MCF-7
SUM 1315
SCR
shRBMS2-1
shRBMS2-2

Figure Legends

Figure S1

Overexpression of RBMS2 increased the sensitivity of MCF-7/DOX cell lines to doxorubicin. RT-qPCR (A) and western blot (B) were performed to confirm RBMS2 overexpression after transfection with lentivirus in MCF-7/DOX cell lines. NC, control; RBMS2, lentivirus overexpressing RBMS2; CCK-8 assay was used to assess the cell viability (C) and DOX IC50 value (D) in MCF-7, MCF-7/DOX and MCF-7/DOX-RBMS2 cells after treatment with different doses of DOX(0,2,10,40,60,80 and 160 μg/mL) of DOX for 24 h. Data were shown as mean ± SD. *p <0.05, **p <0.01, ***p<0.001.

Figure S2

Depletion of p21 could not reverse the sensitization to DOX induced by RBMS2. RT-qPCR (A, B) and western blot (C, D) were performed to verify the effects of p21. CCK-8 kit was used to investigate the DOX IC50 value (E). Data were shown as mean ± SD. *p <0.05, **p <0.01, ***p<0.001.

Figure S3

Overexpression or knockdown RBMS2 could not sensitize breast cancer cells to 5-FU or cisplatin. RBMS2 overexpression and knockdown cell lines were treated with different dosages of 5-FU or cisplatin for 24 h, respectively. CCK-8 assay was used to examine the IC50 value of 5-FU (A, B) and cisplatin (C, D). Data were shown as mean ± SD. *p <0.05, **p <0.01, ***p<0.001.
